# Implementing medical teaching policy in university hospitals

**DOI:** 10.1007/s10459-016-9737-y

**Published:** 2016-11-16

**Authors:** Rik Engbers, Cornelia R. M. G. Fluit, Sanneke Bolhuis, Marieke de Visser, Roland F. J. M. Laan

**Affiliations:** Department for Research in Learning and Education, Radboudumc Health Academy, Radboud University Medical Center, P.O. Box 9101, NL-6500 HB Nijmegen (43), The Netherlands

**Keywords:** Medical teaching policy, Policy implementation, Faculty development, Organizational development, Grounded theory

## Abstract

Within the unique and complex settings of university hospitals, it is difficult to implement policy initiatives aimed at developing careers in and improving the quality of academic medical teaching because of the competing domains of medical research and patient care. Factors that influence faculty in making use of teaching policy incentives have remained underexplored. Knowledge of these factors is needed to develop theory on the successful implementation of medical teaching policy in university hospitals. To explore factors that influence faculty in making use of teaching policy incentives and to develop a conceptual model for implementation of medical teaching policy in university hospitals. We used the grounded theory methodology. We applied constant comparative analysis to qualitative data obtained from 12 semi-structured interviews conducted at the Radboud University Medical Center. We used a constructivist approach, in which data and theories are co-created through interaction between the researcher and the field and its participants. We constructed a model for the implementation of medical teaching policy in university hospitals, including five factors that were perceived to promote or inhibit faculty in a university hospital to make use of teaching policy incentives: Executive Board Strategy, Departmental Strategy, Departmental Structure, Departmental Culture, and Individual Strategy. Most factors we found to affect individual teachers’ strategies and their use of medical teaching policy lie at the departmental level. If an individual teacher’s strategy is focused on medical teaching and a medical teaching career, and the departmental context offers support and opportunity for his/her development, this promotes faculty’s use of teaching policy incentives.

## Introduction

The literature describes different policy initiatives aimed at developing careers in academic medical teaching (AMEE; AERA; Engbers et al. 2013, 2014, 2015; Jaarsma 2012; RCPSC; Schofield et al. 2010; Sorinola et al. 2013; Steinert et al. 2012). Successful implementation depends on faculty making use of these teaching policy incentives.

For most physicians and (bio)medical researchers, developing a career in patient care and/or (bio)medical research is logical because it relates to their primary professional identities as physicians and/or researchers, but in university hospitals they have an extra career path as medical teachers. For most, however, developing a career as medical teachers and making use of teaching policy incentives may appeal to a professional role that is considered secondary to their primary professional identities of physician and (bio)medical researcher. The implementation of teaching policy incentives, in other words, has to deal with the competing demands of patient care and medical research (Bligh and Brice 2010).

There are many general theories on policy implementation that indicate under what conditions a particular innovation will be successful. Grol et al. (2011) give an extensive overview of common implementation theories (see Box 1), which derive from different science fields (e.g., organizational, policy, implementation, and social sciences). Most theories overlap to some extent and have built on each other to develop further. Implementation theories also use different assumptions about human behavior and changing organizations. In general, implementation theories contain factors relating to individual professionals, to social context, to organizational context, and to socio-economic context.

**Box 1:** Overview of different types of implementation theories

Theories on individual professionals Cognitive theories, focusing on individual professionals’ thinking and decision-making Educational theories, on adult development and learning Motivational theories, focusing on behavioral change motivation, such as the theory of planned behavior (Ajzen 1991) and the Triad model (Poiesz 1999)
Theories on social context, including theories on Effective communication to influence behavior Social learning, e.g., the social learning theory (Bandura 1986) The influence of social networks The functioning of teams and leadership
Theories on organizational factors, including theories on Characteristics of effective organizations (e.g., professionalization, centralization, management-intensity) Quality management, aimed at organizational change Learning organizations; human resources development Organizational culture
Theories on socio-economic factors Economic theories, and theories on contracting, such as those on governmental quality standards

Box 1 shows that there are many general implementation theories, but that they often highlight only one aspect, or type of influential factor. Grol et al. (2011) add that for most theories, empirical evidence behind their assumptions about changing human behavior is still limited, and that it is hard to determine what theory is most valid: they can probably all contribute to a better understanding of processes of change and implementation. This means that factors from all different types of implementation theories—individual, social-contextual, organizational, and socio-economic—may relate to the implementation of teaching policy incentives in university hospitals.

In this study, we do not aim to test one particular implementation theory, but rather we want to broadly explore factors that influence faculty in making use of teaching policy incentives in the specific and complex context of a university hospital. Using the factors we find, we want to elaborate a conceptual model for implementation of medical teaching policy in university hospitals, which is offered as stimulus for dialogue and understanding in university hospital departments.

To explore factors that influence faculty in making use of teaching policy incentives, we studied the case of the Radboud University Medical Center (RUMC), which has implemented systems of Teaching Qualifications (TQs), of Principal Lecturer statuses (PLs), and of Subsidized Innovation and Research Projects in Medical Education (SIRPMEs) (Engbers et al. 2013) (see Box 2). We operationalized making use of teaching policy incentives as obtaining a TQ, (J)PL status, or a SIRPME.

**Box 2:** Policy initiatives for medical teaching at the Radboud University Medical Center (RUMC)

(*a*) *The Teaching Qualifications system* The RUMC implemented a system of Teaching Qualifications (TQs), focusing on the medical education setting. TQs help to structure medical teaching careers because they are required for tenure and are considered in appointments in educational positions. The TQ system has four qualification levels Start Teaching Qualification (STQ) Basic Teaching Qualification (BTQ) Extended Teaching Qualification (ETQ) Full Teaching Qualification (FTQ). (Engbers et al. 2013; Engbers et al. 2015)
(*b*) *(Junior) Principal Lecturer statuses* Teachers at the RUMC with a leading role in education can be awarded a (Junior) Principal Lecturer ((J)PL) status (Engbers et al. 2013, 2014). (J)PL statuses involve a financial bonus being granted to the teacher’s department and also serve as criteria for professorship appointments
(*c*) *Subsidized Innovation and Research Projects in Medical Education* Subsidized Innovation and Research Projects in Medical Education (SIRPMEs) are granted annually to encourage innovations and research projects in medical education (Engbers et al. 2013, 2014)

**Box 3:** Financing system for undergraduate medical education at the Radboud University Medical Center (RUMC)

The financing system for undergraduate medical education at the Radboud University Medical Center is characterized by the following points All regular education activities at the RUMC are fully compensated according to a financial distribution model As part of that model, extra money for education can be earned through SIRPME grants, earmarked for educational research or innovation Earmarked (J)PL grants, granted to departments
Without an obligation for departments to spend (J)PL grants on educational research or innovation, they can give financial priority to medical research or patient care. Departments also have to meet financial targets imposed by the EB and may feel obliged to use the money to achieve those targets.

We formulated the following research question: what factors promote or inhibit faculty in a university hospital in making use of teaching policy incentives?

## Methods

### Study design

Given the absence of studies on this type of implementation in the specific context of a university hospital, we chose to use the grounded theory methodology to develop an explanatory theory about factors that affect the implementation of medical teaching policy in a university hospital (Lingard 2014; Lingard et al. 2008; Watling and Lingard 2012).

As is customary in grounded theory, we applied constant comparative analysis (Corbin and Strauss 2008) to qualitative data obtained from interviews. We used a constructivist approach, in which data and theories are co-created through interaction between the researcher and the field and its participants. Theoretical sensitivity was enhanced and theory development was enriched by using the researchers’ own experiences and perspectives (Charmaz 2003, 2009).

To answer our research question, 12 semi-structured interviews lasting 50–60 min were conducted with a purposive sample of educational stakeholders in different departments. A scripted semi-structured interview guide (see “Appendix” for categories and examples of probing questions) was developed by all authors and used for the interviews, pertaining to individual, social-contextual, and organizational factors that promote or inhibit the implementation of organizational policy initiatives for medical teaching. Concepts from different types of implementation theories were included, such as individual motivation, teams, culture, leadership, communication, and organizational structure (Grol et al. 2011).

A test interview with a purposively sampled teacher was conducted to gather feedback on the interview guide and to ensure that the interviews would each be limited to 1 h at most. Because of the information value of the data, we decided to include this interview in our sample for analysis.

To gain representative samples, we first purposively selected sample groups. As we suspected that factors that influence teaching policy implementation might differ between departments with different educational task loads, we divided departments into four categories: <1000 teaching hours; 1000–2200 teaching hours; 2200–10,000 teaching hours; and >10,000 teaching hours a year.

In each of these four categories, we selected one department with low and another one with high use of teaching policy incentives. We did so by computing the average implementation for all four categories, adding up the numbers of TQs, PLs, and SIRPMEs, and dividing them by the total number of departments within that category. Then we divided the four categories into a group of departments with a below-average, and a group of departments with an above-average implementation level for all three policy incentives. This resulted in eight groups.

From each of these eight groups, we then selected one department, making sure to include departments from a range of clinical and non-clinical departments. From these eight departments, we purposively selected our sample group members as data collection and analysis proceeded. We planned the interviews in batches of four, depending on the respondents’ availability.

We targeted both teachers and management (Heads of Department, delegates responsible for medical teaching in the department, and financial managers) because both groups were knowledgeable about the implementation of medical teaching policy in departments but had different responsibilities. We selected teachers and managers from a range of clinical and non-clinical departments (internal medicine, n = 3; cardiology, n = 2; tumor immunology, n = 1; pediatrics, n = 1; dentistry, n = 2; cognitive neuroscience, n = 1; primary and community care, n = 1; biochemistry, n = 1). Because we wanted respondents to be able to speak freely, we interviewed teachers and managers from the selected departments individually.

Participants received a letter explaining the purpose of the interviews and stating that the information they shared would be treated confidentially. The letter also contained a brief topic guide to help participants prepare for the interview. The Ethics Review Board of the Dutch Society for Medical Education granted ethical approval (Nederlandse Vereniging voor Medisch Onderwijs [NVMO]; file number 478).

### Data collection

Data collection and analysis proceeded simultaneously, in an iterative fashion. The principal investigator (RE), who is an educationalist and medical teaching policy advisor, conducted all interviews. As themes emerged from the interviews, new interview probes were added to the interview guide and used in subsequent interviews. New sample group members were each selected purposively as data collection and analysis proceeded. The collection of data ended when saturation of themes was reached, after twelve interviews.

### Data analysis

Interviews were recorded and transcribed verbatim. All transcripts were anonymized and read repeatedly by the first author to ensure accuracy and to permit familiarity. Qualitative Analysis Software Atlas.ti was used to organize and code the data. The first author and a second educationalist and policy advisor (MdV) independently coded all transcripts on a line-by-line basis. Following every third interview, two researchers (RE and MdV) collaboratively carried out a comprehensive analysis of the coded transcripts, creating consensus on a shared codebook. We first extracted and developed concepts from raw data (open coding). Next, we identified relationships between the open codes (axial coding). Finally, we selected a core category and related it to all other categories (selective coding) (Corbin and Strauss 2008).

After six and after nine interviews, two researchers (RE and MdV) facilitated the analytic process by constructing relationship diagrams and affinity maps during two 2-day sessions. Next, the emerging model was discussed by the first author and an experienced educationalist, researcher, and medical doctor (LF). As the model evolved, it was discussed with all authors, including a second experienced educationalist and researcher (SB) and the supervising researcher, who is also a medical doctor (RL). Critical and reflexive feedback from the whole research team was used to improve the rigor of our data collection. While collecting and analyzing data, RE also wrote memos, which became more elaborated, integrated, and focused on specific topics during the process. Collecting memos and diagrams supports a logical and systematic process, grounded in the data (Watling and Lingard 2012).

## Results

Analysis revealed that there were five factors, or themes, that were perceived to promote or inhibit faculty in a university hospital in making use of teaching policy incentives: Executive Board Strategy, Departmental Strategy, Departmental Structure, Departmental Culture, and Individual Strategy.

**Fig. 1:**
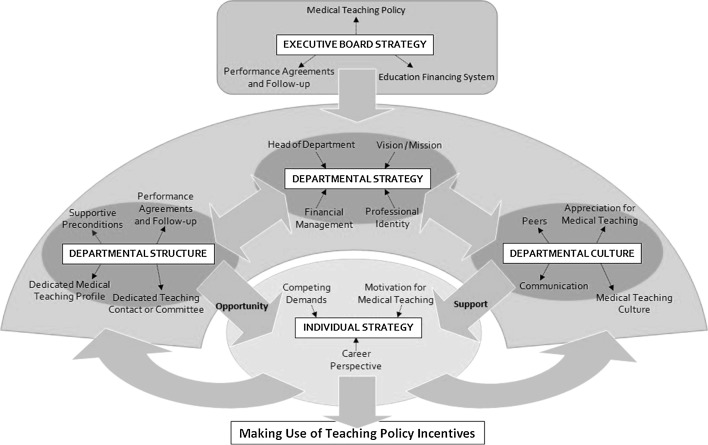
Conceptual diagram representing a model for implementation of medical teaching policy. Individual teachers making use of teaching policy incentives in a university hospital are affected by factors on institutional, departmental, and individual levels

Definitions of components included in these factors and illustrative quotations are presented in Table 1, and a conceptual diagram representing a model for implementation of medical teaching policy is presented in Fig. 1. The five factors and how they were perceived to affect RUMC faculty in making use of teaching policy incentives are explained below.

**Table 1:** Factors perceived to affect faculty use of medical teaching policy

Factors	Components included in definition	Example quote
Executive board strategy	Medical teaching policy Offers career perspectives in medical teaching and cultural change towards medical teaching	"This [the introduction of medical teaching policy] makes a huge difference. At the level of the department’s management, teaching is appreciated considerably more. I can tell…I myself can tell very clearly because up to about six years ago they’d often say to me: that’s all very well, all this teaching, but you’ve got to publish more papers. And I haven’t heard this remark since these PL grants have been around. So now it’s very obvious that teaching actually allows the department to do well and shine. Even up to the point where management is considering who they can launch on this type of career." (P2, 041)
	Education financing system Offering grants for educational innovation can help departments’ focus on medical teaching, but imposing financial targets may negate this effect	“Some of the group’s money is used for educational purposes. But there’s never a lot left. In some years, the targets we were given just about matched the funds we landed. And then you’ve got to pay people’s wages, so there’s little spare cash lying around. Let me see, we got two JPL grants and one JL grant. That’s a nice little sum, over a hundred grand. You could use some of that to appoint a PhD student, but we’ve never had enough left because it had to be spent on wages. Due to the targets we were given. It’s a hard nut to crack, really.” (P2, 234)
	Performance agreements medical teaching and follow-up Educational performance agreements followed up in quarterly meetings between departments and the EB	“Departments were asked to specify what educational activities and innovation activities they’d undertaken with those PL grants, you see. So in other words: the Education Institute was supplying funding in the form of PL grants and they then wanted the departments to tell’em what they were doing with those grants. Well. That’s no longer happening. I don’t know why it was discontinued but it was at some point.” (P12, 052–056)
Departmental strategy	Professional identity The departmental emphasis on patient care, medical research, and medical teaching	“The main duty you’ve got is patient care, simple as that. It may sound trivial but everything else gives way to that. So when you’re very busy, which happens all the time of course, well, teaching and certainly teaching policy just come second.” (P5, 062)
	Financial management Financial efficiency needed for medical research or patient care can affect departments’ strategy	“Money is always a major concern for a department that’s completely dependent on…We get no revenues from courses we give; we get no revenues from patients; we’ve got none of that. And so we’re very dependent on the financial structure, of course. We’ve got a budget of five million, but four million annually is generated from projects. We really need to get cracking with our project proposals. That’s why you need a Principal Investigator (PI) or we just wouldn’t manage as a department.” (P4, 488)
	Head of department The head of department’s attitude towards medical teaching	The head of department must support this [medical teaching careers]. If the head doesn’t, I think all sorts of practical issues tend to gain more and more importance. We try to achieve something and push the limits to do so. But if a head of department doesn’t support it, they won’t be pushing the limits. All of us are now always trying the impossible to make things happen. (P8, 522–526)
	Vision/mission The way in which medical teaching, patient care, and medical research are incorporated into the departments’ vision and mission (is not necessarily head of department’s vision)	Every educational organization must engage in educational development. Reviews are compulsory, aren’t they? So if your education’s not moving forward, you may manage to scrape through one review before it’s damned but you’re likely to be damned first time round. And so you need to have policies in place and you need to have high-profile people who can implement them. (P3, 128)
Departmental structure	Performance agreements and follow-up Educational performance agreements between individual teachers and their departments, including timely goals and follow-up/evaluation	Those with a teaching profile will be held to account in their annual performance interviews: what were their objectives and have they achieved them, yes or no. (P8, 276)
	Supportive preconditions Supporting medical teaching careers in terms of time, money, planning, administration, and balancing workload	This team is being supported by a secretariat. For education. Specifically for education. So they support us in the widest sense of the word: in terms of content, in terms of development, but also financially. So our finances are taken care of and kept in order by this secretariat. (P8, 115)
	Dedicated medical teaching profile Creating career profiles in which medical teaching is a prominent task	And so we’ve chosen to have a kind of dedicated team to deal with education. At present there are four people on this dedicated team, who spend a significant part of their job – so more than 0.2 FTE – on teaching and training. (P8, 061–083)
	Dedicated teaching contact or committee A dedicated contact or committee for medical teaching that focuses on medical teaching policy in the department	We’ve got an Education Committee consisting of four or five people. I’m the chair. We meet once a month. We will also discuss how we can get people to obtain their Basic Teaching Qualification. We really deal with everything relating to educational practice and organization. (P2, 122–124)
Departmental culture	Peers Successful careers in medical teaching and enthusiasm for medical teaching policy generate interest and serve as an example amongst peers	When you see people being involved in TQs and PLs, you’ll be more inclined to get involved yourself than when it’s invisible. It sets an example when you see people being valued and making a career out of it. I believe that’s how it worked for my co-worker X. He’s seen me around and taken it as an incentive that there are rewards for teaching, that it’s not a waste of time. (P2, 210)
	Communication Opportunities in the department to talk about medical teaching and medical teaching policy	We’ve got an Education Committee representing all parts of the department. They prepare retreats. Once a year, we’ll have a retreat on education. There’ll also be one on research and patient care, but there’s a separate one for education. It’ll be dealing with topical issues, but also visions and the future. The new curriculum was a great opportunity, of course, to spend some time together to reflect. Which we always do in… education is always an item on the agenda in our staff meetings. So all in all: the regular meetings, the Education Committee, and the retreats. (P9, 123–127)
	Medical teaching culture Medical teaching and training being part of departments’ core values	We’ve chosen to work in this department because training people is our department’s reason for being. A university is not only a research institution but also a training institute, you know. The goal of this University Hospital is to train doctors and health professionals to work with patients. That’s our reason for being. And that’s why our people should have a passion for working with young people and molding young minds. Whether they’re students, or specialists, or nurses in training, doesn’t matter. The passion must be there. That’s one of our department’s core values. Part of our workplace culture. (P9, 082)
	Appreciation for medical teaching How medical teaching and medical teaching careers are valued compared to careers in patient care and medical research	“There really are people who’d be PLs here but who are considered as, well, underachievers over there [at another faculty, eds] because they’ve only got few publications. They’re considered to be losers. That’s not the case here. Or not anymore, I believe. If you did a lot of teaching to justify your position as it were, that was actually frowned upon as a failure to do research. Things have changed meanwhile.” (P2, 219–221)
Individual strategy	Motivation for medical teaching Individual ambition, being motivated to participate in medical teaching	“Those co-workers that are so committed to teaching just want to get ahead. They actually enjoy being involved in teaching and becoming better teachers.” (P1, 331–347)
	Career perspective Medical teaching valued as a serious career path; medical teaching policy valued as important for career development	“The career perspective is an important one. You’re simply expected to have a Basic Teaching Qualification for many university positions. Here it’s a requirement for associate professors. You can be an associate professor and pursue a career that’s based on education. Or to put it simply: earn more money and get more status.” (P2, 145–212)
	Competing demands The competing demands of medical research and patient care can influence the amount of time and effort put into medical teaching and a medical teaching career.	It’s hard to motivate people to take on more than one core duty or all of them, and we don’t even do any patient care in our department. Of course, education is also something that’ll allow you to shine, but the way it works means you’ve got to choose where you want to shine. That’s how things are. People mainly tend to focus on education and take on research jobs as a hobby or an interest, or the other way around. It’s virtually impossible to do well on both fronts, you know. (P2, 033)

### Executive board strategy

The strategy of the Executive Board of the RUMC is a factor that was perceived by our respondents to affect faculty in making use of teaching policy incentives. The Executive Board at the RUMC is a three-person strategic management board, which includes the dean, who is responsible for education and research. The Executive Board values the quality of medical teaching, which has translated into the development of medical teaching policy, aiming to improve the quality of medical teachers and to create career paths in medical teaching. This policy framework provides an opportunity for departments to focus on the professional development of individual medical teachers. Increasing financial efficiency, patient care, and medical research demands could affect the implementation of medical teaching policy in departments, as could the way this implementation was evaluated or followed up.

#### Medical teaching policy

Respondents indicated that the introduction of medical teaching policy can provide a lever for a cultural shift in favor of medical teaching in departments. The presence of medical teaching policy marks a level of priority and support for medical teaching at an institutional level and provides incentives for departments and teachers to focus on medical teaching careers. As a teacher from a clinical department observed:

Let me put it this way: research has always been considered a top priority in academic circles. It was also very highly valued by the Executive Board. That’s what the Executive Board would use to show off. Patient care even came second for quite some time, I think, in most university hospitals. All that has clearly changed here by putting the patient at the center of care. That’s helped to reduce the gap between research and patient care. The other thing that’s happened is that the Executive Board has raised appreciation for teaching by creating (J) PL positions and by rewarding teaching qualifications. By rewarding these positions in that way, teaching has become more highly valued across the board. That’s very helpful to promote a culture in which education matters. (P8, 418–426)

A manager from a clinical department described how the absence of medical teaching policy at a previous university hospital had prevented people from getting involved and investing in medical teaching:

Promoting factors are: possibilities for people to grow and develop themselves in the field of education. These policy initiatives have helped to define and stake out a clear career in education. Well, I think that’s incredibly attractive to people. I used to work in an academic institution where this wasn’t the case. And then you see that people don’t tend to go for a career in education and don’t invest in it either. So I think that one of its main effects is that it helps people to build a full career. That’s one of its main effects, I believe. In addition, it makes policy visible; it makes the goals of the entire hospital visible. It provides a particular structure that helps people to channel their activities. (P9, 313)

The way policy initiatives for medical teaching have been designed and supported were reported to affect how individual teachers make use of them. The transparency of the (J)PL procedure was reported to stimulate the submission of applications. A clinical teacher said:

I think the PL positions are a very positive development. Because I feel they simply offer appreciation to those who want to dedicate themselves to teaching. And because they’re transparent: How do I qualify? What are the criteria? There’s a list you can check of who’s become a PL. It’s an option you can choose to apply for every year. This openness, I think, is a very positive development. A boost. (P7, 288)

The path to obtaining a TQ, however, was perceived to be more barrier-strewn, more rigid, and less well facilitated. A respondent from a clinical department said this caused people to lose their motivation to obtain a TQ:

As a coordinator, I can explain very well to teachers, including those in my own program, that I think it’s important for them to acquire such expertise because you learn a lot when you enroll in a Teaching Qualification program. Which is all very well, but when a course proves to be fully booked for the whole of next year, well, I’m flabbergasted. It’s a pretty rigid system still. That’s a shame. I’ll lose those people who are really motivated to qualify because it’ll take forever for them. It’s a shame. (P7, 058)

The same applied to the way SIRPME rounds are organized. Respondents experienced a lack of feedback and follow-up after they had applied for a SIRPME. A clinical teacher commented:

I think it’s great that we have such a scheme now. But it’s completely unclear, not just to me personally but to our department, what applications get accepted and why. You may get very favorable comments in the first and second round and still be rejected. So why do you get it? And why don’t you? Who manages to get their project funded? What’s the allocation system? It’s all completely obscure. This is really discouraging for your next application. (P7, 292)

## Education financing system

The consistent implementation of medical teaching policy is influenced by the financing system for undergraduate medical education used at the RUMC (see Box 3). On the one hand, respondents reported that PL grants provided a financial incentive for departments to focus on medical teaching and for faculty to make use of teaching policy incentives. According to one Head of Department:

The financial side of things is very important, of course, for money is a way to make things happen. So this PL grant makes it possible for someone to be a PL and to spend time on education. (P9, 177)

On the other hand, the same Head of Department reported that financial targets imposed by the Executive Board (see Box 3) affected the department’s financial decision-making priorities, inhibiting its focus on medical teaching:

This means: making choices. Well, that’s what we’re trying to do. You can’t do everything. We can’t do everything, but the Executive Board does expect us to achieve a certain turnover, so we’re caught in a squeeze there. If it was up to us, we’d say: ‘Let’s get rid of some consultation hours.’ But we can’t do that because we wouldn’t be making enough money. So this is a problem. (P9, 108)

## Educational performance agreements and follow-up

For medical teaching policy to be rooted in departments, a certain degree of follow-up is necessary to make sure departments get and stay involved. The departmental numbers of TQs, PLs, and SIRPMEs, however, appear not to be discussed in the quarterly appraisals of departments, while patient care and medical research are always on the agenda. If departmental involvement in medical teaching policy is not evaluated, this could negatively affect their implementation strategy with regard to medical teaching policy. As a manager from a clinical department put it:

I feel that relations between the Executive Board and the department have always been minimal in the matter of education. We try to make it a high-priority issue so we tell them: ‘Dear Executive Board, why don’t you just make education an item on your agenda in one of your quarterly meetings once a year and ask the departments what they’re up to.’ Because, you know, we’ve got information we can feed them. I think things may change with the introduction of the new curriculum, so I’m wondering what’ll happen in these meetings. Will the Executive Board just take note of a department’s report? Or will they discuss what the report implies, so they can have a genuine dialogue with this department? (P11, 336)

### Departmental strategy

The department’s strategy was perceived by our respondents to affect the implementation of medical teaching policy. The extent to which departmental strategy focuses on medical teaching is influenced by the following characteristics:

#### Professional identity

Participants pointed out that departments differed in their emphasis on patient care, medical teaching, or medical research. If medical teaching was described to be a department’s main professional identity, this helped the implementation of medical teaching policy in this department. According to a Head of Department:

We’re mainly an education department. So it matters a great deal to us that we meet Teaching Qualification standards. (P3, 094)

A surgical clinician described the emphasis of his department as being fully focused on patient care. Making use of medical teaching opportunities, let alone aspiring to a career in medical teaching, does not fit well with this type of professional identity:

Because patient care is still always everyone’s favorite. If you asked me what I’d most like to become, I’d say: Principal Clinician. Suits me like a glove. And I think 80 % of our staff would say the same if you asked them. (P5, 261–365)

Another respondent mentioned the presence of typical research departments, whose strategic focus is fully on medical research:

Research is still very prominent in some departments, but it’s a hell of a job now to keep it that way. So this means you need to invest a lot of time and energy, even if a lot of people involved don’t have tenured positions. And so, if you have to make this choice for research, as a person or as a department, or if you want to retain staff. If you want to prioritize research to education. That’s a true research department. They’re still around and always will be. (P8, 438–446)

### Financial management

The financial efficiency needed for medical research and patient care may affect departments’ medical teaching strategies. A manager from a clinical department underlined this by bringing up the increasing call for cost-effective patient care:

Yes, patient care is changing a lot, mainly because of the introduction of regulated market forces in the healthcare sector. This means that efficiency has become more important than it used to be. And so business management is also playing an increasingly important role here. (P12, 093–095)

#### Head of Department

The Head of Departments’ support was reported to be conditional for a strategy focusing on medical teaching. A teacher from a non-clinical department reported a positive example:

The fact that the head of department allows people to do this instead of saying: ‘No, no, you’ve got to do more research.’ It does encourage people. Or it gives people the opportunity to do this. (P2, 180)

A negative example was given by a respondent from one of the surgical departments, who sensed that the Head of Department lacked commitment to medical teaching:

Well, perhaps our department’s head is not committed enough to education and considers it a bit of an obligation. Care is close to his heart, you see. (P6, 227)

#### Vision/mission

As a counterpart to financial frameworks determining departmental strategy, a Head of Department believed in a vision of matching personal ambitions to departmental needs in medical teaching as well as in research and patient care:

What are the department’s needs? And what suits people’s career development? There needs to be a good match between these two. And then there’s the financial context. As a department, we’ve decided we would never adjust our policy to financial considerations. Content is number one, and our story had better convince the Board to allow us to continue. I think that’s also what matters when people must decide in a couple of years’ time whether our department is to stay or go. Money won’t be their first concern but rather what we do and who we are. So that’s our top priority. (P9, 119)

### Departmental structure

In addition to the extent to which medical teaching careers are part of the departmental strategy, the extent to which departments have set up a structure to support the development of medical teaching careers was perceived to affect the implementation of medical teaching policy. The presence of such a supportive structure was considered not only to be a result of the departmental strategy, but also to influence the departmental strategy at the same time. The following elements of departmental structure were mentioned:

#### Performance agreements and follow-up

If a teacher wants to pursue a career in medical teaching, and the departmental strategy allows for it, performance agreements and timely follow-up can provide the structure to actually move forward. A respondent from a clinical department observed:

Choices and agreements. This means we always make agreements on objectives. So there are preconditions but also objectives in our annual performance interviews. This is what the education committee does in the department. It’s what I do with individuals in the dedicated team. And it’s what heads of sections also do with individuals. So we’ll say to them: ‘Look, you’ve got this education profile, but what are your objectives?’ And we also agree on a timeframe. So far, all those objectives have actually been achieved. I’m really pleased about that. So we make annual agreements with each other on content, form, objectives, and the itinerary. I do performance interviews with people not just as a coach but also as head of the education team and I’ll ask them: ‘What is it you want? What are your choices in education and what do you want to go for? What conference can you attend to profile yourself? How can you achieve JPL or PL status?’ Etcetera. (P8, 570)

#### Supportive preconditions

The presence of a number of supportive preconditions can help teachers to make use of teaching policy incentives. A manager from a clinical department described how a balanced workload can be facilitated:

You can have three duties and do them all on the same day. But you can also say: ‘I’ll take three days and do one duty on one day and another duty the next day.’ This creates more balance and continuity. You could even do the same with a weekly schedule. Say someone has an intensive teaching schedule over a number of weeks but is not senior consultant or ward supervisor in those same weeks. (P9, 199)

A senior teacher from a clinical department said that departments in general have not invested enough in a supportive structure, resulting in fewer teachers who obtained their TQ in a timely fashion:

I think departments have not done much to organize such things for a group of specialists. In our department, at least, it is a pretty solitary process. They agree in their performance interview that they’ll obtain a teaching qualification; then they find out what to do, who to see, and where to go. All on their own. And when they’ve done all that and their next performance interview comes round, and then you have to conclude that nothing’s happened. I think that’s a shame. (P7, 066)

The absence of support was also reported to inhibit the use of medical teaching policy in this surgical department:

Well, and perhaps they haven’t taken care of the preconditions properly, in the sense of time, money – yes, money, of course – and support. (P6, 365)

#### Dedicated medical teaching profile

Participants reported that the demands of patient care and medical research made it challenging to perform in medical teaching. As a Head of Department said:

We believe you can’t be a major league player with three of your core duties. (P9, 119)

One way to deal with performance pressure on three fronts is to create dedicated profiles in which two duties are combined. People will still be asked to contribute to all key duties, but they will be accountable for the results in their chosen profile. This way, dedicated medical teaching profiles can be created, offering both time and opportunities for a career in medical teaching, including the use of medical teaching policy. A teacher from a clinical department reported:

Two years ago we decided that each faculty member should get, or take, two duties. One of those is patient care and then you have another one. That may be management, or research, or teaching. So we have also had dedicated teaching staff since that time. (P8, 065)

#### Dedicated teaching contact or committee

A dedicated contact or committee in the department to discuss matters relating to medical teaching policy and medical teaching careers was considered important for the implementation of medical teaching policy. As a teacher from a clinical department put it:

Yes, it’s an absolutely important core duty. For patient care, we have several dedicated contacts: the head of the inpatients’ department and the head of the outpatients’ department. We also have one for research and so we certainly need one for education. If you haven’t got one, I think that things get too fragmented and patchy. (P7, 149)

### Departmental culture

The extent to which the departmental culture is focused on medical teaching was perceived by our respondents as affecting the implementation of medical teaching policy. Departmental culture and departmental strategy and structure are perceived to mutually influence each other. Departmental culture was reflected in the following characteristics:

#### Peers

The presence of peers who obtained (J)PL status, a TQ, or a SIRPME triggered enthusiasm for medical teaching policy in both non-clinical and clinical departments. One participant said:

I think it’s all about enthusiasm. No, it’s all about a department’s choice. That’s one. And two, it’s the enthusiasm of people who’ve chosen to do it and who are willing to go for it. So the pioneers and the initiators. They’re the ones who blazed the trail, before it was appreciated and rewarded by the department and the hospital. Then people take notice. They may never have thought about education as an option, but they may have picked it up because of them. (P8, 362)

#### Communication

Communicating medical teaching policy initiatives in departments was mentioned as a good way to generate structural awareness of medical teaching careers. A non-clinical teacher mentioned the following ways in which medical teaching policy was discussed regularly:

So let me summarize the structure properly: we have a management team, who meet twice a week. Then we have committees who prepare things that are submitted to this central management team. And then we have staff meetings for all faculty, once every three months, where we discuss things like education policy. (P2, 134)

Teachers cannot make use of teaching policy incentives if they are not aware of the possibilities. One clinical teacher indicated that the opportunity to apply for SIRMPEs had never been communicated by the Head of Department:

As a regular teacher, I’ve never heard the Head of Department mention it. But the Head of Department may well have discussed it in the Council of Professors, and then it‘ll end up with the departmental heads. (P1, 159)

Obtaining (J)PL statuses, TQs, or SIRPMEs were cause for celebration in some departments. Communication of these successes was reported to have a positive effect on the development of medical teaching careers. The head of one clinical department described the spin-off effect of success this way:

Well, we tend to celebrate these things. They get mentioned on our team’s website. They get a lot of exposure. People themselves are proud of it, and so are we as a department. And the PLs have an inspirational effect, you know, they in their turn stimulate other people to become PLs. So yes, it helps a lot in realizing a career in education. (P9, 287)

#### Medical teaching culture

Some participants mentioned the presence of a medical teaching culture in their departments. In a positive teaching culture, teachers do not need to be stimulated to make use of teaching policy incentives. The head of one clinical department even described a passion for medical teaching as a core value of the department:

It’s crystal clear that people in this department are passionate about teaching. Absolutely. So I don’t even need to stimulate them in any way, and I actually feel I shoudn’t have to. (P9, 082–086)

A non-clinical teacher described the cultural change towards medical teaching that took place over the past decade:

You can tell there’s a culture of achievement, encouraging people to improve themselves, make portfolios, etc. It encourages people to take a look at each other’s educational activities, to discuss educational activities more, how you can improve them. It’s becoming more of an item. I think if you compare this with ten years ago…well, no one would’ve said: it doesn’t matter what you do in teaching, but it was never a topic of discussion. Now it is, particularly amongst staff themselves. (P2, 194–198)

#### Appreciation for medical teaching

The shared departmental conviction that medical teaching is as important as medical research was felt to be an incentive to make use of teaching policy incentives. One clinical teacher reported that the explicit appreciation for medical teaching careers had helped to elevate the position of medical teaching towards that of medical research:

There’s still a difference of course. The Principal Investigator position is slightly higher valued than that of Principal Lecturer, but the gap has become much smaller. I think that’s because we’ve positioned them as equal. Taking this position has led to a change of culture. Teaching wasn’t valued at all when I first arrived here, but now it’s greatly appreciated. And the people who take the lead and do it are also valued and rewarded for their effort. Not only in terms of time but also in terms of prestige. So this benefits everyone, including me. (P8, 184–188)

### Individual strategy

Respondents reported that the extent to which an individual teacher’s strategy was focused on medical teaching affected his or her use of medical teaching policy. The extent to which an individual teacher’s strategy is focused on medical teaching is influenced by the following characteristics:

#### Motivation for medical teaching

We found that, for teachers, being motivated to engage in medical teaching provided a stronger focus on medical teaching when it comes to personal strategy, goals, and ambitions. Participants indicated that teachers who are passionate about medical teaching and who have a drive to participate and to perform well in medical teaching are more likely to be applying for a TQ, a SIRPME, or (J)PL status as part of their personal strategy. A teacher from a clinical department commented on the passionate attitude towards medical teaching in his department:

Teaching is part of my job and I happen to like it. It’s all in a day’s work of course, that you just do it, even if the gains are not so clear. Well, in our department everybody likes teaching and developing materials. I guess that we would’ve had the same attitude if we hadn’t had those PL grants. (P10, 066–130–312)

A teacher from another non-clinical department described motivation for medical teaching as promoting the use of medical teaching policy in this way:

There are certainly those who enjoy teaching and developing teaching materials. And the ones who like it anyway, they are the ones who are willing to seize the opportunity to get a certificate, let’s say. As a basic teaching qualification. (P2, 147)

### Career perspective

Introducing medical teaching policy as part of the Executive Board’s strategy was reported to provide incentives for teachers to focus on a medical teaching career. Participants reported that the extent to which medical teaching was valued as a serious career path and the extent to which individual strategy was aimed at pursuing a career in medical teaching influenced people’s choice to make use of teaching policy incentives. As a non-clinical teacher reported about people’s motivation to apply for a TQ or (J)PL status:

We’ve got some staff in our department, often young people, who need to put together a career plan. In the end, all of them want to be associate professors, so they all need to choose either research or teaching specializations. When they choose teaching, they must aim to obtain JPL or PL status, that’s part of the procedure. (P3, 136)

A respondent from a clinical department described the relation between people’s willingness to make use of teaching policy incentives and their individual ambition to pursue a medical teaching career as follows:

If you have personal ambitions in the field of teaching, I think that you’re prepared to make investments and take those things in your stride that aren’t immediately useful. You just do them or you won’t get there. But if you’re otherwise inclined and you just have to…well, time is just too precious.” (P7, 112)

#### Competing demands

The competing demands of medical research and patient care influence the extent to which an individual teacher’s strategy is focused on medical teaching and a medical teaching career. A participant from a non-clinical department said about the competing demands of medical research:

A lot of people want a job at a university because they’re interested in doing research. And that’s how their performance is held to account: their career, being Principal Investigator, number of publications, etc. Everything else is in competition with that. So everything that takes time and trouble limits what you can do in the field of research. And when I look around, I feel that’s a pretty big obstacle. Oh well, obstacle may be saying too much. It’s a major competitor, let’s say. (P2, 031)

A teacher from a clinical department mentioned the department’s emphasis on patient care and the amount of time it took up:

Whenever my colleague’s not there, I’m supposed to take over those teaching duties. But I tend to pass the bucket to someone else because there’s always the pressure of patient care. So time is indeed a constraint, in particular because patient care is considered to be more important than teaching. (P5, 062)

### Interaction between individual strategy and departmental context

The mutual interaction between teachers’ individual strategy and their departmental context was clear from our interviews. Departments offer opportunities and support for individual teachers, whose strategies and choices in their turn influence departmental strategies, cultures, and structures (see Fig. 1). The two quotes below illustrate the interaction between individual and departmental interests, and how creating a supportive environment may induce individual teachers to make use of teaching policy incentives:

Well, there are so many things going on; you just need to make your policies very clear at the end of the day: what is it you want? Where do you want to go? And then try to achieve that. So this means there’s got to be some give and take. And this means that you try to create a kind of protective environment for people who are very keen and very young so they can actually achieve it. What we’ve done, for instance, as you know, is to get postgraduate medical students to obtain basic teaching qualifications. We’ve got two of those now. One of them has almost finished and is to be a fellow here. So he’s going to pursue a career in education. And if he stays here, that’ll be his second profile. (P8, 131)

You need to have a certain scope or a certain framework for people to be able to develop themselves. And what people bring to this framework is their own creativity. And this creativity in its turn requires a certain structure. So yes, you need to have an interplay between these things, I think. It’s a bit like networking actually. (P9, 165)

## Conclusions and discussion

We found factors that promote or inhibit individual teachers in a university hospital in making use of teaching policy incentives on three different levels: the individual level, the departmental level, and the institutional level. The individual and departmental levels mutually influenced each other; the institutional level influenced the departmental level.

### The individual level

The decision to make use of teaching policy incentives is ultimately made at the individual level. We conclude that the more individual teachers’ strategies are focused on medical teaching, the more likely it is that they will obtain a TQ, (J)PL status, or a SIRPME. We found that the extent to which individual teachers’ strategies are focused on medical teaching is influenced by their motivation to engage in it, by the priority they give to it in comparison to medical research and patient care, and by their aspiration for a career in medical teaching.

Most of the factors we found to affect individual teachers’ strategies and their use of medical teaching policy lie at the departmental level, which is plausible because this is the level where teaching policy is realized. Moreover, when teachers are asked what factors affect their use of medical teaching policy, they probably focus first on the immediate social context of their departments. We conclude, therefore, that the extent to which the departments’ structure, strategy, and culture are focused on medical teaching influences the extent to which individual teachers’ strategies are focused on medical teaching.

### The departmental level

A departmental structure that focuses on medical teaching has the following characteristics: creating dedicated medical teaching profiles; offering supportive preconditions that help to balance workload; appointing a dedicated teaching contact or committee; communicating (successes in) medical teaching policy; and structural follow-up on performance agreements in medical teaching.

The departmental strategy and vision are influenced by the Heads of Department. Financial efficiency and the competing demands of patient care and medical research may affect the extent to which medical teaching is part of the departmental strategy and vision.

A departmental culture that accommodates appreciation for medical teaching also encourages individual teachers to focus their strategy on medical teaching. We encountered such a culture mainly in departments where medical teaching and medical teaching careers are valued more equally to careers in patient care and medical research. A departmental culture that is supportive to medical teaching careers is characterized by interaction between all peers and management. Aspects that appear to promote the use of teaching policy incentives in departments include communication opportunities about medical teaching, medical teaching policy, and successes obtained in medical teaching policy.

The crux of our model lies in the mutual interaction between individual teachers and their departmental context. In short: if the individual teachers’ strategy is focused on medical teaching and a medical teaching career, and the departmental context offers support and opportunity for this development, this promotes the use faculty make of teaching policy incentives.

### The institutional level

At the institutional level, the Executive Board’s medical teaching strategy and policymaking may affect the departments’ focus on medical teaching careers. If departments make insufficient use of medical teaching policy without this having any consequences, this may undermine the implementation of medical teaching policy. Financial targets imposed by the Executive Board could also negate the positive effect of educational innovation grants on departments’ focus on medical teaching.

### Relation to other theories

Our results reflect the notion that, as Grol et al. (2011) mentioned, all different types of implementation theories can contribute to a better understanding of processes of change and implementation. Our conceptual model relates to combined aspects of theories on factors relating to individual behavior, social context, and organizational context.

At the individual level of our model, teachers’ individual strategy relates to factors from cognitive theories (deciding on career paths and dealing with competing demands) and educational and motivational theories (the motivation to learn and develop as a medical teacher).

At the interface between the individual and the departmental level, our model shows the influence of social context on individual behavior. This resonates with implementation theories focused on both individual and social-contextual factors, such as the theory of planned behavior and the social learning theory, which posit that social norms influence behavior (Ajzen 1991; Bandura 1986). The large transparent arrows in our model indicate the reciprocal relations between individual staff and their departments. More specifically, our model shows relations between individual strategy, departmental opportunity and support, and faculty making use of teaching policy incentives. This is broadly in line with the Triad model, which assumes that people will engage in a specific behavior if their motivation, perceived capacity, and perceived opportunity to execute this behavior are sufficiently high (Poiesz 1999).

We found that the more the departmental structure, strategy, and culture offer support and opportunities for individual teachers to develop, the more likely teachers are to take ownership of their own professional development, and this finding also relates to the work of Fuller and Unwin (2004). Their theory on expansive and restrictive learning environments states that expansive learning environments stimulate workforce development. Expansive learning environments offer, for example, opportunities for employees to pursue knowledge-based as well as competence-based qualifications, have a recognized status as a learner, and have access to career progression and extended job roles. Billet (2001) also mentions the importance of what he calls workplace affordances. Having access to teaching policy incentives aimed at career progression as a medical teacher, and receiving departmental support obtaining a TQ, SIRPME, or (J)PL status resemble these expansive features.

At the departmental level in our model, moreover, communication about teaching policy incentives, peers, and Heads of Department are presented as factors that influence individual teachers’ behavior. This is in line with implementation theories on the influence of effective communication, the functioning of teams, and leadership.

Our conceptual model for implementation of teaching policy incentives resembles the model for weaving scholarship of teaching and learning into institutional culture (Williams et al. 2015). Williams et al. (Williams et al. 2015) describe macro-, meso-, and micro-levels in higher education institutions. The macro-level sets the strategic direction at an institutional level, much like the executive board strategy in our model. The meso-level (middle management, Heads of Departments etc.) interprets key issues, much like the departmental strategy on medical teaching and on medical teaching policy. At the micro-level (individual faculty), social networks are important for the emergence of an academic culture. In our model, departmental strategy, structure, and culture are closely linked. They are directly shaped and influenced by individual teachers, and are decisive for successful implementation of teaching policy incentives. Both models underline the importance of effective communication, of sustained support from management, and of a departmental culture serving as a supportive social network.

Our model is also in line with notions from theories on learning organizations and organizational culture. The mutual interaction between departmental strategy, structure, and culture displayed in our model fits well with notions from Marquardt (Marquardt 1996), who considers organizational strategy, structure, culture, and vision to be key dimensions for learning and organizational development. In our model, we assume that a shared vision influences departmental strategy and individual behavior.

In short, for research on implementation of teaching policy incentives in the rich and complex context of university hospitals, a single theory is not sufficient. Multiple aspects of implementation theories on the individual, social, and organizational levels must be taken into account.

### Strengths and limitations

A limitation of this study is that our data were gathered in one university hospital and that our specific context might have led to findings that are not applicable to other situations.

However, the findings in this study do provide a new understanding of the implementation of medical teaching policy in university hospitals. Our results are in line with several implementation theories on the influence of individual, social-contextual, and organizational factors. We expect, therefore, that our findings may inform other studies.

### Implications for practice and further research

Our results suggest that departments, both clinical and non-clinical, should offer a supportive environment, structurally, strategically, and culturally, to enable individual teachers’ strategies to be focused on medical teaching. Two clinical departments created dedicated medical teaching profiles, and several non-clinical and clinical departments offered supportive preconditions to facilitate teachers to take career steps in medical teaching. If we take these departments as an example, this could be beneficial for other departments struggling with the competing demands of medical research and patient care. Discussing medical teaching policy and careers during staff meetings, for example, may also contribute to a departmental climate that focuses more on medical teaching. Our conceptual model for the implementation of medical teaching policy in university hospitals is offered as stimulus for dialogue on further improving the quality of medical teaching in university hospital departments.

The results of this research support the idea that the Executive Board should follow up on the implementation of medical teaching policy in quarterly meetings between departments and the Executive Board. Educational performance agreements addressed in those meetings should include the award of PL grants and the results of granted SIRPMEs. The Executive Board could use the findings from this study to stimulate faculty to make use of teaching policy incentives in departments.

Because our data were gathered in one university hospital, more information on what factors promote or inhibit the implementation of medical teaching policy in departments at other university hospitals would help us to establish a greater degree of accuracy on this matter. With data from other university hospitals, we could test and refine our conceptual diagram representing a model for the implementation of medical teaching policy. Future research should also be undertaken to explore how the factors we found to affect the implementation of medical teaching policy are related, as we have not yet measured these relations.

Further studies including members of the Executive Board and different departments could also reveal factors on a departmental or perhaps individual level that affect the Executive Board’s strategy.

Lastly, our model does not show the possible effects of obtaining a TQ, (J)PL status, or a SIRPME because we only asked our interviewees about the factors that promoted or inhibited individual teachers in a university hospital in making use of teaching policy incentives. It is conceivable that the actual use faculty make of teaching policy incentives affects individual teachers’ strategy and their motivation for medical teaching, as some studies have shown relations between policy initiatives and individual teachers’ motivation for medical teaching (as an outcome measure for the quality of medical teaching) (Engbers et al. 2014, 2015), and other outcomes such as educational leadership (Newman et al. 2015). Further research, therefore, could usefully explore the effects of the use faculty make of teaching policy incentives. This may add another arrow to the model.

In conclusion, at the interface between individual staff and their departments, individual ambitions to pursue a career in medical teaching can be fostered and supported. Our model, focusing on the reciprocal relationship between individual staff and their departments, may help in aligning personal ambitions and departmental goals and strategies, and to promote the implementation of medical teaching policy.

## Appendix

See Table 2.

**Table 2:** Guide for semi-structured interview (categories with examples of probing questions)

What comes to mind first when I ask: “What factors promote or inhibit faculty in a University Hospital in making use of teaching policy incentives?”
Practical matters? Is medical teaching facilitated in terms of time? Time-pressure? Why? Other tasks? Enough time for preparation? Practical matters like class schedules? Task size/involvement in medical teaching? Insufficient financial compensation? Why? Compared to what? What could solve this?
Competing demands of patient care and medical research? Patients’ interest? Separated time-investment patient care and medical teaching? Medical research is more important. How can you tell? How could this change? Career perspectives? We find medical teaching just as important as …
Individual/personal opinions and circumstances? Personal interest/ambition? (Dis)interest in medical teaching: How come? How could this change? Familiarity with teaching policy incentives? How? Through what channels? Personal circumstances that put work under pressure?
Departmental management? Teacher: what are promoting or inhibiting factors in departmental management? Departmental vision of medical teaching? How was it formed? Is it shared? Assessment interviews? Relationships? Consequences? Policy plan? Education paragraph? Does management appreciate teachers’ involvement in education? How does it show? Does the executive board appreciate departments’ and teachers’ involvement in education? How does it show? Is use of teaching policy incentives actively encouraged? How are teachers informed about and involved in teaching policy incentives? Informal talk about teaching policy incentives? Awareness of/importance of/support for JPL, TQ, SIRPME? Why? (teachers, colleagues, management) Do TQs/JPLs/SIRPMEs affect teaching quality?
Department’s interest in medical teaching? Colleagues’ interest in medical teaching? Department’s appreciation for medical teaching, for TQs, JPLs, SIRPMEs? Is everyone informed? Are people interested? Do teachers talk about medical teaching (policy) in their departments? With whom? Does management facilitate or encourage conversations about education?
Teaching Policy Incentives in themselves: suitable or not? Do they promote quality of education or an educational climate? Do they arouse interest in medical teaching (policy)? How do they relate to patient care policy and medical research policy? What other teaching policy incentives would you like to see?
Facilitating factors outside the department (other groups, cultures, communities of practice)? Collaboration with other departments/teachers? Appreciation outside the department? Do teachers talk about medical teaching (policy) outside their departments?
Any other factors we did not discuss?
